# Quantitative method for analysis of six anticoagulant rodenticides in faeces, applied in a case with repeated samples from a dog

**DOI:** 10.1186/s13028-018-0357-9

**Published:** 2018-01-17

**Authors:** Kristin Opdal Seljetun, Elin Eliassen, Ritva Karinen, Lars Moe, Vigdis Vindenes

**Affiliations:** 1 Department of Companion Animal Clinical Sciences, Faculty of Veterinary Medicine, Norwegian University of Life Sciences (NMBU), P.O. Box 8146 Dep, 0033 Oslo, Norway; 2 Division of Environmental Medicine, Norwegian Poisons Information Centre, Norwegian Institute of Public Health, P.O. Box 4404 Nydalen, 0403 Oslo, Norway; 3 Department of Forensic Sciences, Division of Laboratory Medicine, Oslo University Hospital, P.O. Box 4450 Nydalen, 0424 Oslo, Norway; 4 Institute of Clinical Medicine, Faculty of Medicine, University of Oslo, P.O. Box 1171 Blindern, 0318 Oslo, Norway

**Keywords:** Brodifacoum, Bromadiolone, Coumatetralyl, Difenacoum, Difethialone, Flocoumafen, Half-life, Pharmacokinetics, Rodenticide poisoning, Serum

## Abstract

**Background:**

Accidental poisoning with anticoagulant rodenticides is not uncommon in dogs, but few reports of the elimination kinetics and half-lives in this species have been published. Our objectives were to develop and validate a new method for the quantification of anticoagulant rodenticides in canine blood and faeces using reversed phase ultra-high performance liquid chromatography–tandem mass spectrometry (UHPLC–MS/MS) and apply the method on a case of anticoagulant rodenticide intoxication.

**Results:**

Sample preparation was liquid–liquid extraction. Six anticoagulant rodenticides were separated using a UPLC^®^ BEH C_18_-column with a mobile phase consisting of 5 mM ammonium formate buffer pH 10.2 and methanol. MS/MS detection was performed with positive electrospray ionization and two multiple reaction monitoring transitions. The limits of quantification were set at the levels of the lowest calibrator (1.5–2.7 ng/mL or ng/g). The method was successfully applied to a case from a dog accidentally poisoned with anticoagulant rodenticide. Coumatetralyl and brodifacoum concentrations were determined from serial blood and faecal samples. A terminal half-life of at least 81 days for coumatetralyl in blood was estimated, which is longer than previous reported in other species. A slow elimination of brodifacoum from the faeces was found, with traces still detectable in the faeces at day 513.

**Conclusions:**

This study offers a new method of detection and quantification of six frequently used anticoagulant rodenticides in canine faeces. Such drugs might cause serious health effects and it is important to be able to detect these drugs, to initiate proper treatment. The very long elimination half-lives detected in our study is important to be aware of in assessment of anticoagulant rodenticide burden to the environment.

## Background

Anticoagulant rodenticides (AR) are used worldwide in pest control. The first generation AR includes warfarin, chlorophacinone, diphacinone and coumatetralyl that were developed in the 1950s. Increasing resistance in rodents led to the development of second generation compounds [1, 2]. These long-acting anticoagulant rodenticides include brodifacoum, bromadiolone, difenacoum, difethialone and flocoumafen, which are far more toxic and lethal for strains of rodents resistant to the first generation rodenticides [2].

The AR produce their anticoagulant effect by inhibition of vitamin K_1_ epoxide reductase. This prevents regeneration of active vitamin K_1_ and thus impairs formation of vitamin K_1_ dependent clotting factors II, VII, IX and X, and proteins C and S in the liver [3]. The anticoagulant effect is mainly due to depletion of factors II and X [4]. In the dog, the plasma half-lives of factors II and X are 41 and 17 h, respectively [5]. After the depletion of the already circulating clotting factors, spontaneous coagulopathy develops. Clinical signs after ingestion of AR are expected to develop after about 3.5 days, which represents minimum two half-lives of clotting factor II [4].

Ingestion of AR is not uncommon in dogs and other non-target animals and has been documented over several years [6–9]. We do not know, however, how many dogs in a population are exposed to these rodenticides during their lives. In 2014, a survey of prevalence of previous exposure to AR in diseased dogs was undertaken at the Norwegian University of Life Sciences (NMBU) [10]. Liver samples were taken from all the dogs that were necropsied during 6 months’ time, irrespective of the cause of death, illness or clinical signs. Rodenticides were detected in the liver in one in five dogs (20%) of the 63 dogs included in the study. In 8% of the necropsied dogs more than one type of AR were present. The source of the rodenticide in these dogs could not be determined.

The liver is the organ with the most significant accumulation of AR, and the major route of elimination is through bile to the faeces [11–13]. The long duration of action is explained by their enterohepatic circulation and high lipid solubility [14, 15]. In an experiment done in foxes with multiple doses of bromadiolone, residues persisted in the liver even when bromadiolone was no longer detectable in plasma [16]. The excretion in faeces continued throughout the study period of 31 days and was still present at the end of the study.

Detection of AR requires rapid, sensitive and specific methods. Warfarin and its metabolites are regularly analysed by gas chromatography or high-performance liquid chromatography, but owing to larger mass and lower volatility of some of the AR, liquid chromatography–mass spectrometry has been considered a more suitable method [17]. Several analytical methods for detection of AR have been published [18]. There are no published methods for determining concentration of AR in faeces from dogs. In addition, there is sparse information describing the toxicokinetics of coumatetralyl in blood in the canine species.

The main objective of this study was to develop an analytical method for analysis of six AR in faeces. We used the method to determine the elimination time of coumatetralyl in blood and faeces after an acute poisoning of AR in a dog; a case history is presented.

## Case history

A 7.2 kg, 6-month-old intact female Dachshund presented to the University Animal Hospital at NMBU after an ingestion of 1.5 block of AR nicked from the owners pocket. The information of the product or AR dose were not available. Within one and a half hour following ingestion, the dog was given apomorphine to induce vomiting, which revealed some large pieces of rodenticide. The dog was given activated charcoal and referred to a veterinary clinic for measurement of prothrombin time (PT) and activated partial thromboplastin time (aPTT) at 48 and 72 h after exposure. Due to a misunderstanding, the blood samples were not examined at the clinic, but sent to an external laboratory and the prolonged coagulation was not discovered.

Five days after exposure, the dog returned to the University Animal Hospital. Clinical signs included lethargy, weakness, tachycardia, weak pulse, pale mucous membranes, tachypnea and dyspnea. The initial coagulation profile showed a markedly prolonged PT of 51 s and aPTT of 131 s. Vitamin K_1_ was administered orally and symptomatic treatment was initiated. The clinical condition improved gradually over the next 2 days and the dog’s PT and aPTT levels returned to normal. The dog was discharged to her owners’ care on day 9, and the vitamin K_1_ antidote treatment continued for 50 days after ingestion. This improved the clinical condition, but is not expected to affect the kinetic curve of AR [19]. The dog remained healthy with a complete resolution of clinical signs throughout the study period.

## Methods

### Sample collection and storage

Faecal samples were collected from the poisoned dog in dark plastic bags or plastic containers after natural defecation on the same day as the blood collection. Samples were maintained at − 20 °C. Within a few weeks, the samples were lyophilized to dryness. The sample residues were analyzed at the laboratory at the Department of Forensic Sciences, NMBU.

Blood for analyses of AR was collected into vacuum tubes containing sodium fluoride as preservative and potassium oxalate as anticoagulant. Blood samples were frozen (− 20 °C) shortly after collection and stored until analyses.

Blood for analyses of aPTT and PT was obtained in a vacutainer tube containing sodium-citrate (3.2%). The blood was analyzed within 2 h of collection at NMBU by a Coag Dx Analyzer (IDEXX Laboratories Europe B.V., The Netherlands).

Blood and faecal samples for determination of AR in our dog were obtained 6, 7, 11, 18, 22, 24, 32, 39, 50, 64, 93, 121, 204, 422, 470 and 513 days after ingestion. Corresponding measurements of PT and aPTT were made in the acute phase of the poisoning.

### Reference substances and chemicals

**Fig. 1:**
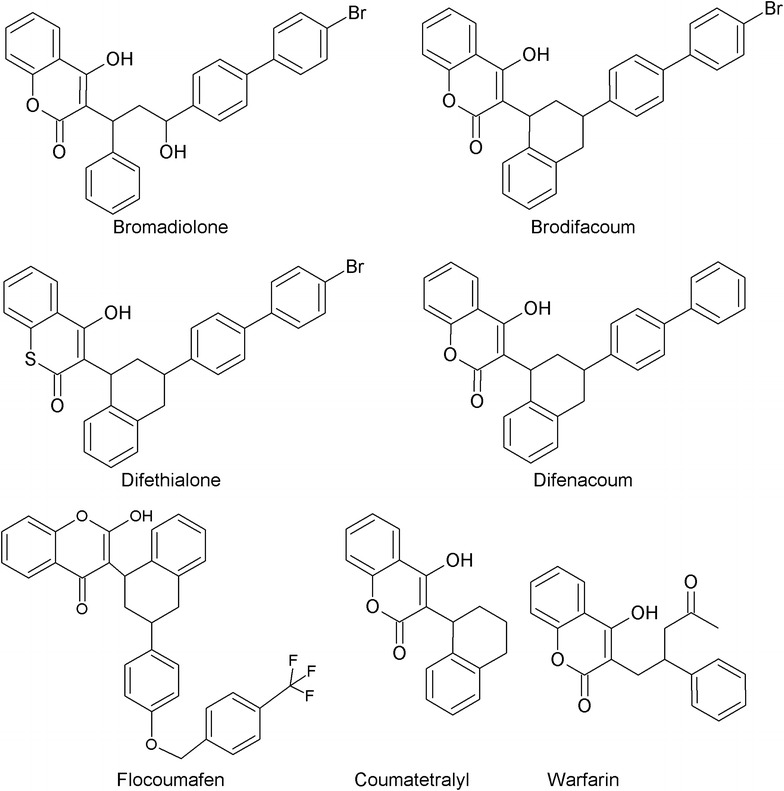
Molecular structures of the compounds and warfarin

Brodifacoum, bromadiolone, difenacoum, flocoumafen were supplied by Fluka Chemika (Sigma-Aldrich Norway AS, Oslo, Norway), difethialone and coumatetralyl by Dr. Ehrenstorfer (Dr. Ehrenstorfer GmbH, Augsburg, Germany). Figure 1 presents the compounds’ molecular structures. Warfarin-d5 (internal standard) was purchased from Chiron AS (Chiron AS, Trondheim, Norway). Ethyl acetate and dichloromethane were obtained from Chemi-Teknik as (Oslo, Norway). Methanol (LC–MS Chromasolv^®^), acetonitrile (ACN), ammonium formate and heptane (99%) were purchased from SIGMA (Sigma-Aldrich Norway AS, Oslo, Norway). Type 1-water (18 MΩ-cm) was obtained from a Milli-Q A10 water purification system (Millipore, Bedford, MA, USA). Human whole blood was supplied by Blood Bank at Ullevål University Hospital, Oslo, Norway, and the blank dog faeces samples were collected from other healthy dogs by the authors.

Stock solutions of the analytes were prepared separately in ACN, and working standard solutions for brodifacoum, bromadiolone, difenacoum, flocoumafen, and coumatetralyl were prepared in ACN from the stock solutions at seven concentration levels. Working standard solutions for difethialone was prepared separately because of the lower concentration of the reference substance solution. Calibration samples were prepared from whole blood or faeces spiked with working standard solutions. The concentration ranges are shown in Table 1. Quality control (QC) samples were prepared independently at three concentration levels.

**Table 1:** Validation parameters of six anticoagulant rodenticides

Compound	Calibration range (ng/mL or ng/g)	Blood		Faeces		QC-sample conc.	Blood					Faeces				
		Mean R^2^	RSD (%)	Mean R^2^	RSD (%)		Within-day precision RSD (%)	Between-day precision RSD (%)	Bias (%)	ME%	RE%	Within-day precision RSD (%)	Between-day precision RSD (%)	Bias (%)	ME%	RE%
Coumatetralyl	1.5–731 (0.0050–2.5 µM)	0.999	0.061	0.998	0.24	2.3 88 585	4.0 3.6 6.0	7.9 8.7 11	− 3.5 5.2 − 1.8	90 93	10 15	2.8 5.7 6.5	15 13 15	− 3.6 1.6 8.4	109 96	69
Bromadiolone	2.6–1319 (0.0050–2.5 µM)	0.998	0.21	0.998	1	4.2 158 1055	5.8 5.7 7.5	17 14 17	16 13 − 4.1	100 97	44 52	15 17 16	16 18 13	− 7.7 − 3.1 − 11	63 56	32
Difenacoum	2.2–1111 (0.0050–2.5 µM)	0.997	0.33	0.999	0.10	3.6 133 889	9.1 2.2 8.9	13 15 15	5.5 7.8 6.1	101 97	43 87	13 14 15	18 17 15	− 5.3 − 6.1 − 11	62 60	26
Flocoumafen	2.7–1356 (0.0050–2.5 µM)	0.997	0.41	0.995	1.1	4.3 163 1085	8.0 3.0 6.4	14 14 15	2.7 6.6 − 4.6	95 92	80 90	11 9.0 6.3	19 17 19	− 5.5 − 2.8 − 14	45 32	18
Brodifacoum	2.6–1309 (0.0050–2.5 µM)	0.998	0.37	0.999	0.11	4.2 157 1047	8.6 3.1 9.1	15 14 11	8.4 6.2 − 3.1	93 90	61 78	9.2 9.3 6.4	19 18 19	− 1.7 − 2.1 − 18	32 32	25
Difethialone	2.7–1349 (0.0050–2.5 µM)	0.997	0.37	0.998	0.17	4.3 164 1079	8.9 6.4 13	14 12 17	16 12 3.1	86 81	69 84	10 11 5.1	17 11 16	1.3 0.8 − 9.4	31 27	22

### Blood sample preparation

Sample preparation for calibrators and controls was performed by adding 50 µL of each working standard solutions in ACN to an aliquot of 100 µL whole blood. 100 µL ACN was added to the unknown samples (100 µL). 50 µL of the internal standard (0.078 mg/L in Type 1 water) was added to all samples followed by immediate agitation on a Multitube vortexer. 100 µL borate buffer pH 11 and 1.2 mL ethyl acetate/heptane mixture (4:1 *v/v*) were added and the samples were agitated for 10 min followed by centrifugation at 4500 rpm (3900×*g*) at 4 °C for 10 min. The organic layer was transferred to a clean 5 mL glass tube, dried under N_2_ (nitrogen gas) at 40 °C, reconstituted with 100 µL of methanol/Type 1 water mixture (20:80 *v/v*) and shaken well before transferring into auto sampler vials.

### Faecal sample preparation

The fecal samples homogenized and exact aliquots of 100 mg were weighed in using a precision weight (XS-precision weight, ©Mettler-Toledo International Inc., UK). Preparation of the calibrators and QC-samples were performed by adding 50 µL of each working solutions to the blank faeces samples. To the case samples, 100 µL ACN was added. To all samples, 50 µL internal standard and 400 µL borate buffer pH 11 were added followed by immediate agitation on a Multitube vortexer. 1.0 mL ACN was added followed by agitation. 1.0 mL dichloromethane was added and the samples were mixed for 10 min using a blood mixer followed by centrifugation at 4500 rpm (3900×*g*) at 4 °C for 10 min. The thin, upper messy layer was carefully removed; and the dichloromethane phase was transferred to a clean glass tube, dried under N_2_ at 40 °C, and reconstituted with 100 µL of methanol/Type 1 water mixture (20:80 v/v), shaken, and centrifuged before transferring into auto sampler vials.

### Analysis

The samples were analyzed in on a Waters ACQUITY UPLC-system (Waters Corporation, Milford, MA, USA), applying an Acquity UPLC^®^ BEH C_18_-column (2.1 mm × 50 mm, 1.7 µm particles, Waters Corporation, Milford, MA, USA) using gradient elution with a mobile phase consisting of 5 mM ammonium formate buffer pH 10.2 (A) and methanol (B). The column temperature was held at 65 °C and the mobile phase flow rate was 0.5 mL/min. The gradient profile was: 10% B in 0.00–1.50 min, 30% B in 1.50–1.80 min, 58% B in 1.80–1.81 min, 60% B in 1.81–3.50 min, 60% B in 3.50–3.52 min, 100% B in 3.52–4.00 min, 100% B in 4.00–4.50 min, and 10% B in 4.50–4.51 min. A linear curve profile for the change in mobile phase composition was used. Run time was 6.00 min and the injection volume 3 µL.

**Fig. 2:**
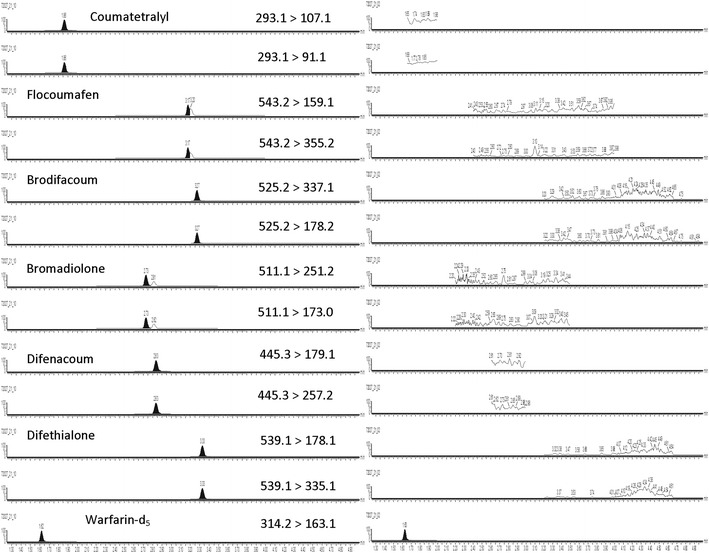
The chromatograms of the lowest quality control sample and the blank sample with the internal standards

Positive electrospray ionization (ESI+) MS/MS detection was performed on a Xevo TQS triple quadrupole mass spectrometer from Waters (Milford, MA, USA), using two multiple reaction monitoring (MRM) transitions for each analyte and the internal standard. Data acquisition, peak integration, and calculation were interfaced to a computer workstation running MassLynx 4.1 software. The MRM transitions monitored, along with the respective cone voltage and collision energy values, and retention times for the analytes, are listed in Table 2. The chromatograms of the lowest QC sample and the blank sample with the internal standards are shown in Fig. 2.

**Table 2:** Multiple reaction monitoring transition ions

Compound	RT (min)	MRM transitions (m/z)	Cone voltage (V)	Collision energy (eV)
Coumatetralyl	1.86	*239.1* *>* *107.1*/91.0	40/30	32/28
Bromadiolone	2.73	*511.1* *>* *251.2*/173.0	26	24/42
Difenacoum	2.83	*445.3* *>* *179.1*/257.2	30	32/22
Flocoumafen	3.17	*543.2* *>* *159.1*/335.2	28	42/24
Brodifacoum	3.27	*525.2* *>* *337.1*/178.2	34/55	34/55
Difethialone	3.33	*539.1* *>* *178.1*/335.1	36	32/22
Warfarin-d_5_	1.62	*314.2* *>* *163.1*/256.0	24	14/22

Transitions in italics font were used for quantification

### Method validation

Quantitative results were obtained by integrating the peak height of the specific MRM chromatogram in reference to the integrated height of the internal standard. A 2nd order calibration curve (y = ax^2^ + bx + c) was used for quantification because of the wide concentration range (Table 1). Origin was excluded and a weighing factor 1/x was used. Limits of quantification (LOQ) were set at the level of the lowest calibrators, signal-to-noise ratios were above 10. Within-day (n = 6) and between-day variations (n = 6) were determined by analyses of spiked human whole blood and blank faeces samples at three different concentration levels for all compounds. Faecal analyses were performed using 3–6 parallels for each sample. Extraction recovery and matrix effect were studied using the method developed by Matuszewski et al. [20]. For this study, five dog blood samples and faeces samples were spiked at two concentration levels for all compounds. Extraction recoveries for blood samples were studied at two concentration levels and at one level for faeces samples.

## Results

The calibration curves were evaluated and mean values of R^2^ were above 0.995 for all compounds in both blood and faeces (Table 1). The levels of the lowest calibrator (1.5–2.7 ng/mL blood or ng/g faeces) fulfilled the criteria for LOQ for all compounds. Precision and accuracy, determined as bias, are shown in Table 1, and was within ± 20% for all compounds. For blood, no pronounced matrix effects were seen, while for faeces ion suppression was observed for all compounds except for coumatetralyl. Extraction recovery was likewise lower from faeces than from blood.

**Fig. 3:**
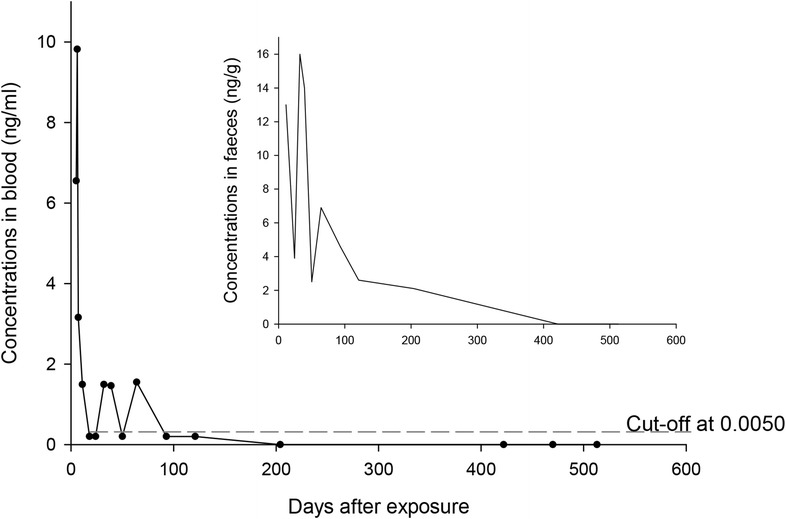
Concentrations of coumatetralyl in blood (ng/mL) and faeces (ng/g)

The elimination curves for coumatetralyl in blood and faeces were estimated (Fig. 3). The initial distribution phase could not be established in this case, as the analysis of blood was first performed 6 days after ingestion. However, the elimination from day 6 to day 11 indicates a first phase with an estimated half-life of 1.8 days, which indicates an initial α-elimination phase. At 18 days after ingestion, the blood concentration was below LOQ but continued to vary around and below this concentration for 4 months after ingestion. The last positive blood sample was seen 64 days after ingestion.

The corresponding faecal analyses of coumatetralyl were performed using 3–6 parallels for each sample. Relative standard deviations (RSD) were between 4 and 41%, with an average of 25%, for all the samples from our case. Large visible plant material, etc. were removed before sample preparation, but the variability in sample aliquot content will always be large in this type of samples. This partly explains the relatively large relative standard deviations of the analysis between the concentrations found for the sample aliquots. The first elimination phase in faeces could not be accurately determined as the first samples were taken 11 days after ingestion. The second elimination phase from day 64 to 422 gives an estimated eliminations half-life of at least 81 days. Coumatetralyl was still detectable in faeces 204 days after ingestion, which demonstrates a considerably longer presence in the faeces compared to blood.

**Fig. 4:**
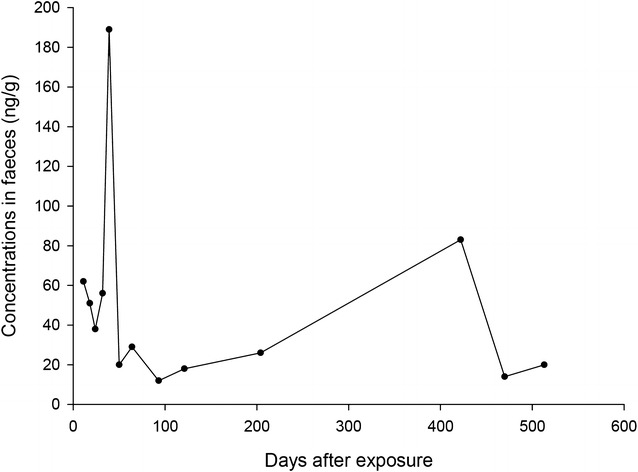
Concentrations of brodifacoum in faeces (ng/g)

Detectable levels of brodifacoum were found in blood throughout the study period. Since only one of the concentrations where above LOQ, these results are not presented in Fig. 4. Corresponding analyses of brodifacoum in faeces demonstrated relatively high levels throughout the study (Fig. 4).

## Discussion

We developed a novel method for analysis of six AR in faeces from dogs, and detected coumatetralyl and brodifacoum in blood and faeces and a very long elimination in the faeces. Accidental poisoning with AR is not uncommon in dogs, but few reports of the elimination kinetics and half-lives are published.

### Elimination of coumatetralyl in faeces

The enterohepatic circulation and major route of elimination through the faeces support analyzing AR in faeces as a measurement of the residues in the body. Previous studies have found the highest concentration of AR in the liver, followed by kidney, muscle and fat [13, 21]. The lowest concentration was detected in blood [13]. In our case concentrations of coumatetralyl in faeces increased from day 24 to day 32 (Fig. 3). One explanation for this second peak could be a new exposure to AR, but we consider this unlikely since the corresponding concentrations in blood displayed only trace amounts of coumatetralyl and no clinical signs of exposure. Analytical error has been explored; and six parallels of the sample from day 32 were run, and the concentration in the following sample continued to be elevated. An explanation is biological variabilities between samples from the same animal [22–25]. A more probable explanation of the second peak is enterohepatic recirculation. Bile is released from the gallbladder shortly after ingestion [26]. Our samples were not collected at the same time after meals. The dog was in her first estrus at day 32 and 39. Both estrogen and progesterone have extensive enterohepatic recirculation and are shown to decrease bile flow [27, 28], but the impact on the excretion of anticoagulant rodenticides is not known.

The faecal coumatetralyl concentration of 2.1 ng/g at day 204 suggests a substantial storage in the liver. Our analyses give an estimated terminal half-life of at least 81 days in this dog. A previous study in rats determined the elimination half-life of coumatetralyl in the liver to 55 days [29]. A stipulation of the elimination half-life in the liver of mice is 15.8 days [30] and 18.9 days in red deer (*Cervus elaphus scoticus*) [31]. Our results indicate that coumatetralyl might be present in the liver for more than 7 months after a single ingestion in dogs, depending on the amount ingested. As no samples were taken between days 204 and 422, we could not verify the elimination more precisely.

### Detection of coumatetralyl in blood

There is limited toxicokinetic data available for coumatetralyl. Our case demonstrates a rapid initial α-phase in whole blood with concentrations decreasing from 9.8 to 1.5 ng/mL from day 6 to day 11, representing a half-life of 1.8 days. A terminal phase with slower elimination followed, until coumatetralyl was not detectable at day 204. This indicates a biphasic elimination, suggesting a two-compartment model, in accordance with studies from mice [30]. Compared to studies of other first-generation AR, our results indicate that coumatetralyl is detectable in the blood of dogs for a longer period compared to other investigated species. In rats, a single dose of chlorophacinone was completely excreted within 4 days [32]. A potential interaction on coumatetralyl elimination from brodifacoum cannot be excluded, but only trace amounts of brodifacoum was detectable in the blood throughout our study. The coumatetralyl dose ingested will affect the detection time.

### Sources of brodifacoum

The source of brodifacoum in our case could not be determined. The dog had not showed any signs of illness prior to this ingestion and the owners were unaware of previous AR exposure. The trace amounts in blood indicated no recent, large ingestion. There are no AR products legally available in Norway, which contains both coumatetralyl and brodifacoum [33]. A previous exposure of small amounts of brodifacoum could have taken place. Another explanation of the small amounts of brodifacoum found in this young dog may be through exposure to a resistant or sublethally poisoned rodent. Resistance to second-generation AR is observed in the brown rat (*Rattus norvegicus*) and house mouse (*Mus musculus*) in several European countries [34, 35]. In Germany, sublethally contaminated mice are detected in large areas around baiting stations [36]. Fisher et al. demonstrated an excretion of up to 19.4% of the ingested AR in the faeces of rats before death at day 4–6 [37]. Exposure to faeces from poisoned animals may be another origination of brodifacoum. Brodifacoum poisoning by a fecal–oral route has been suggested in one human case after a chronic accidental exposure [38]. The extent of this impact requires further investigation.

### Faecal elimination of brodifacoum

We demonstrated high brodifacoum residues in the faeces throughout the study (Fig. 4). Extraction recovery for brodifacoum was 25%, which increases the risk of false negative results in our analyses. Our LOQs were set at the levels of the lowest calibrators (1.5–2.7 ng/g), which is below 3 µg/kg dry matter faeces in a previous study in foxes [16]. Our validation procedures yielded a satisfactory result for blank faeces samples, and precision and accuracy was within ± 20%. We believe this substantiates our method as precise, in spite of the low extraction recovery. After an initial reduction, brodifacoum concentration increased from day 24 to day 39, which corresponds to similar increase in concentration of coumatetralyl. Equivalent explanations as for coumatetralyl is probable for this peak. A second peak in faecal concentration of brodifacoum was seen at day 422, with corresponding concentration in blood displaying trace amounts. The owners were unaware of any new exposure and had removed all rodenticides from their property after the initial poisoning. The dog had not displayed any clinical signs of poisoning during these 7 months, but as no samples were collected between day 204 and 422, re-exposure to AR cannot be excluded.

No canine studies of hepatic half-life of brodifacoum could be found, and we propose to use of serial faecal levels to determine AR liver residues. Brodifacoum was still detectable at the conclusion of the study at day 513. Studies of the second-generation AR brodifacoum in rats after a single oral dose indicate biphasic elimination from the liver, with an estimated half-life of 150–350 days [32]. A single dose of brodifacoum in possums produced high liver concentrations at the time of sacrifice at 254 days [39]. An experiment with a single oral dose of brodifacoum in sheep demonstrated detectable levels in the liver at the end of the trial at day 128, but below the limit of detection in the faeces at day 32 [40]. This comparatively short elimination time could be explained by the limit of detection in faeces of 0.05 mg/kg (equivalent to 50 ng/g), compared to our study with a LOQ of 1.5 ng/g. A species difference between the ruminants and the monogastric dog may also be a contributing factor.

### Detection of brodifacoum in blood

Few studies have reported half-life of brodifacoum in blood from dogs. A study with four dogs and administration of brodifacoum for 3 consecutive days, suggested a terminal half-life of 6 ± 4 days, revealing a two-compartment model and biphasic elimination [19]. A non-compartment model is suggested in one report, with a median plasma half-life of 2.4 days in seven poisoned dogs [41]. As the source and time of ingestion of brodifacoum were unknown in our case and only trace amounts were detectable in blood during the 513 days, we were not able to establish the elimination half-life. Our data suggests, however, a more prolonged half-life compared to previous studies.

### Coumatetralyl poisoning

Coumatetralyl is classified as a first generation anticoagulant that requires multiple ingestions in order to exert its effect [42]. In our case, a single ingestion produced a severe poisoning. However, the trace amounts of brodifacoum detected in the blood and faeces at the time of ingestion may be a contributing factor to the severe effect of coumatetralyl in this case. The correlation between residues in the liver and sublethal effects in the animal is poorly described [43]. Riley et al. [44] showed a significant association between death in mange-infested bobcats and secondary anticoagulant exposure, suggesting that small exposures to AR lead to increased susceptibility to other diseases. Another study [45] did not find association between exposure to AR and immune suppression in cats. Other studies in rats have demonstrated severe poisoning after a single exposure of coumatetralyl [46, 47]. Different susceptibility to coumatetralyl between species has been suggested in several previous studies [42, 48]. Chopra et al. [47] described 50.5 mg/kg body weight of coumatetralyl in rats to be lethal to all the Indian mole rat (*Bandicota bengalensis*), but ingestion of 176.5 mg/kg was necessary to achieve equivalent effect in the common house rat (*Rattus rattus*). Species variation in susceptibility to coumatetralyl has also been demonstrated between Malaysian house rat (*Rattus rattus diardii*) and ricefield rat (*Rattus argentiventer*) [49]. There is a lack of data of the toxicity of coumatetralyl in dogs, but this species is suggested to be the most sensitive of the non-target mammals to coumatetralyl after a single ingestion [50]. One report suggested the lowest dose with effect on the coagulation to be 1 mg/kg in dogs, while similar effect is achieved after 5 mg/kg in cats [51]. The ingested dose of coumatetralyl in our case was unknown, as the product was not identified and the dog vomited some of the AR a short time after ingestion.

### Limitations

Faeces contain varying concentrations of AR, due to the inhomogeneity of the sample aliquots. This will further affect extraction recovery and the concentration of AR. Due to ethical considerations, studies of AR in non-target animals such as dogs are unacceptable in many countries. Naturalistic studies like ours will thus provide valuable contribution to this field.

## Conclusions

We have developed a new method for the quantitative determination of six anticoagulant rodenticides in blood and faeces from dogs, by using UPLC–MS/MS. This analysis of AR in faeces offers a rapid, precise and non-invasive technique to monitor rodenticide exposures and adds value to diagnosing intoxication. The assay was successfully applied to a case of accidental rodenticide poisoning in a dog with analyses of faeces and blood. The faecal analyses of coumatetralyl revealed an estimated terminal half-life of at least 81 days in dogs. Brodifacoum was still detectable at the conclusion of the study at day 513, and displayed a prolonged half-life compared to previous studies. To our knowledge, this is the first report of a method for analysis of anticoagulant rodenticides in the faeces from dogs.

## Abbreviations

ACN: acetonitrile
aPTT: activated partial thromboplastin time
AR: anticoagulant rodenticides
LOQ: limits of quantification
MRM: multiple reaction monitoring
N2: nitrogen gas
NMBU: Norwegian University of Life Sciences
PT: prothrombin time
QC: quality control
RSD: relative standard deviations
UHPLC–MS/MS: ultra-high performance liquid chromatography–tandem mass spectrometry
